# The catalytic role of the M2 metal ion in PP2Cα

**DOI:** 10.1038/srep08560

**Published:** 2015-02-24

**Authors:** Chang Pan, Jun-yi Tang, Yun-fei Xu, Peng Xiao, Hong-da Liu, Hao-an Wang, Wen-bo Wang, Fan-guo Meng, Xiao Yu, Jin-peng Sun

**Affiliations:** 1 Key Laboratory Experimental Teratology of the Ministry of Education and Department of Biochemistry and Molecular Biology, Shandong University, School of Medicine, Jinan, Shandong, China; 2 Qilu Hospital of Shandong University, Jinan, China; 3 Shandong Provincial School Key laboratory for Protein Science of Chronic Degenerative Diseases, Jinan, Shandong, China; 4 Department of Physiology, Shandong University, School of Medicine, Jinan, Shandong, China; 5 Department of Human Biology, University of Toronto, Toronto, Ontario, Canada; 6 Yangtze Delta Region Institute of Tsinghua University, Zhejiang, China; 7 Provincial Hospital affiliated to Shandong University, Jinan, Shandong, China

## Abstract

PP2C family phosphatases (the type 2C family of protein phosphatases; or metal-dependent phosphatase, PPM) constitute an important class of signaling enzymes that regulate many fundamental life activities. All PP2C family members have a conserved binuclear metal ion active center that is essential for their catalysis. However, the catalytic role of each metal ion during catalysis remains elusive. In this study, we discovered that mutations in the structurally buried D38 residue of PP2Cα (PPM1A) redefined the water-mediated hydrogen network in the active site and selectively disrupted M2 metal ion binding. Using the D38A and D38K mutations of PP2Cα as specific tools in combination with enzymology analysis, our results demonstrated that the M2 metal ion determines the rate-limiting step of substrate hydrolysis, participates in dianion substrate binding and stabilizes the leaving group after P-O bond cleavage. The newly characterized catalytic role of the M2 metal ion in this family not only provides insight into how the binuclear metal centers of the PP2C phosphatases are organized for efficient catalysis but also helps increase our understanding of the function and substrate specificity of PP2C family members.

Reversible protein phosphorylation is one of the fundamental regulatory mechanisms that modulate various biological processes, including but not limited to growth and differentiation, immune response, metabolism and neuronal activities^1^. The PP2C phosphatases (the type 2C family of protein phosphatases; or metal-dependent phosphatase, PPM) constitute a distinctive phosphatase family, the members of which are widely expressed across many different species and are known to be essential genes in abiotic stress responses in plants or cell cycle regulation in yeast^2^,^3^,^4^,^5^,^6^. In *Homo sapiens*, the PP2C family has at least 17 members that are pivotal players in many physiological or pathological processes. For example, protein phosphatase 1D (PPM1D, PP2Cδ or Wip1), the product of the human PPM1D gene, contributes to oncogenic transformation that plays vital roles in heterochromatin silencing and the cell cycle^7^,^8^. PPM1D mutations are linked to brain stem tumors, breast cancer and ovarian cancer^8^,^9^,^10^. PP2C domain-containing protein phosphatase 1K (PPM1K or PP2Cκ) is an essential metabolic modulator of branched-chain amino acids, and PPM1K mutations are associated with the maple syrup urine disease^11^,^12^. Protein phosphatase 1G (PPM1G or PP2Cγ) controls transcriptional elongation, DNA damage and RNA splicing^13^,^14^,^15^; PH domain leucine-rich repeat-containing protein phosphatases (PHLPPs) that dephosphorylate protein kinase B (PKB, also known as Akt) and protein kinase C (PKC) are cardinal regulators of cell survival and growth^16^,^17^. PKB (Akt) is a kind of serine/threonine-specific protein kinases and plays a key role in multiple cellular processes such as cell proliferation, apoptosis, cell migration and transcription.

Despite their distinct functions in cells, all PP2C family members have conserved active sites and share similar catalytic mechanisms. The active site of PP2C is located at a cleft between two central β sheets, and it harbors a binuclear metal ion site required for catalysis^18^. The two hexa-coordinated metal ions are bridged by the carboxylate side chain of D60 and a water molecule (Fig. 1a). Reducing the pK_a_ of the water molecule by using the two coordinated metal ions enables the oxygen to attack the phosphorous atom of the substrate nucleophilically via an S_N_2 mechanism during catalysis^18^,^19^. Although the essential roles of the binuclear center in PP2C family member activity have been confirmed by several biochemical and crystallographic analysis, two central, mutually linked questions regarding the catalysis of PP2C family members remain unanswered. Why do these phosphatases need two metal ions? What is the contribution from each metal ion to the catalysis and function of PP2C family members? The unelucidated roles of each metal ion during catalysis are partially explained by the lack of an efficient strategy to specifically remove or exchange the metal ion from one specific site^4^,^20^,^21^. In our recent studies, we discovered that cadmium is a potent and specific inhibitor of PP2C family phosphatases that acts by binding to the M1 site of the binuclear metal center (Fig. 1a)^19^. The specific reduction in the negative charge of the M1 site coordinated residue D441 in PPM1G altered the metal inhibition profile^19^. These results suggested that the M1 metal ion is required for catalysis and determines the metal ion inhibitory profile of PP2C phosphatase. By contrast, the contribution of the M2 site metal ion during substrate hydrolysis has not been investigated. Therefore, we set out to delineate the catalytic role of the M2 metal ion by specifically mutating its surrounding residues. The mutations of conserved D38 in PP2Cα (the product of the PPM1A gene) to Ala or Lys impaired the protein's cellular functions and decreased its phosphatase activity. Moreover, crystallographic analysis uncovered that these D38 mutants disrupted Mn^2+^ binding at the M2 metal ion site. Our study provides the exact roles of the M2 metal ion during catalysis, which will help not only in understanding the catalytic properties of PP2C family members but also extend our knowledge about metal-mediated metalloenzyme catalysis.

## Results

### The M2 metal ion binding site in the PP2C family

**Figure 1:**
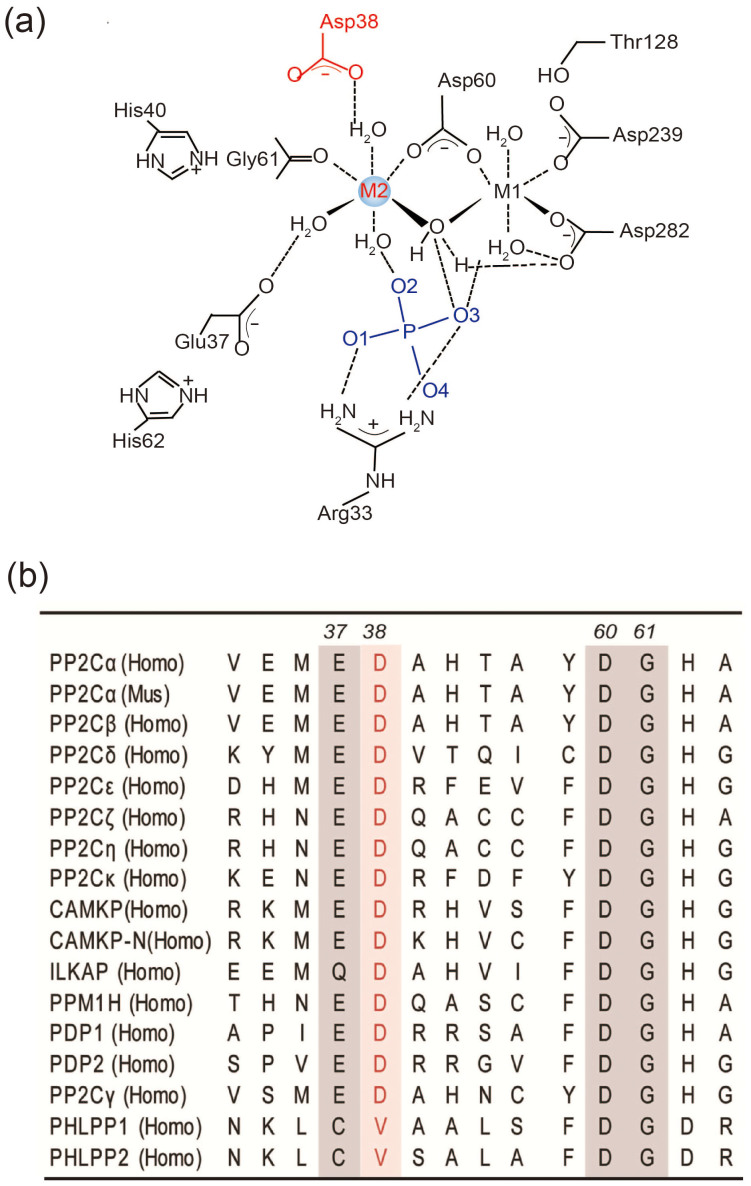
A structural diagram of the PP2Cα active site and sequence alignment of the M2 coordinated residues for the PPM family members. (a) A schematic representation of the active center of human PP2Cα. (b) A multiple amino acid sequence alignment of PP2C family members from *Homo sapiens*. The 38^th^ residue of PP2Cα and the corresponding amino acid residues of other members are marked in red. Residues involved in the M2 metal ion coordination are shadowed.

The PP2Cα is the prototype PP2C family phosphatase for which the catalytic mechanisms and cellular functions have been well characterized. Therefore, we chose PP2Cα as a model for investigating the catalytic role of the M2 metal ion. In the crystal structure of PP2Cα, the M2 metal ion is directly coordinated to the primary chain carbonyl of the Gly61 and the side-chain carboxylate group of Asp60 (Fig. 1a). Both Asp60 and Gly61 are invariant residues among all PP2C members in *Homo sapiens*. Asp60 coordinated to both the M1 metal ion and the M2 metal ion, and an Asp60 mutation to Ala or Asn decreased the phosphatase activity more than 30-folds (Fig. 1b). In addition to these direct interactions, the M2 metal ion of PP2Cα also indirectly interacts with Glu37 and Asp38 through water-mediated hydrogen bonds (Fig. 1a). Neither Asp38 nor Glu37 is involved in direct M1 metal ion coordination, and both residues are conserved among most PP2C family members (Fig. 1b and Supplementary Fig. S1). In comparison with Glu37, Asp38 is more conserved along a broader scope of different phosphatases (Supplementary Fig. S1). Therefore, we altered the side chain properties of Asp38 to see whether it can provide a specific tool for investigating M1 metal ion functions.

### The D38 mutants impair PP2Cα-regulated signaling pathways in cells

We made a D38A mutation in PP2Cα, which eliminated the D38 side chain and abolished the indirect interaction between D38 and the M2 metal ion. To reverse the negative charge of the D38, we also made a D38K mutant to see the mutating effect.

**Figure 2:**
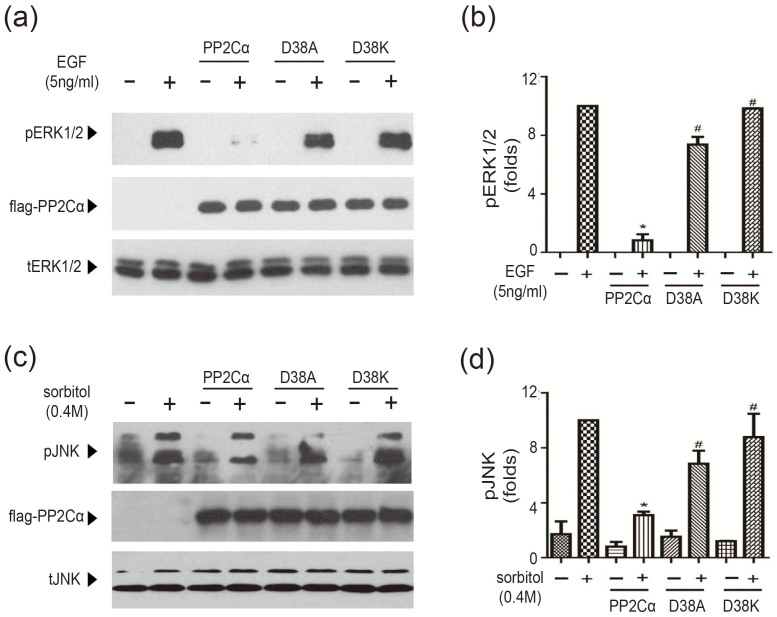
D38A and D38K mutants impaired the cellular functions of PP2Cα. (a) The cellular effects of PP2Cα-WT, D38A and D38K mutant overexpression on EGF-induced ERK phosphorylation in HEK293 cells. The HEK293 cells were cultured in DMEM containing 1% penicillin/streptomycin, 10% fetal bovine serum and 25 mM glucose at 37°C and 5% CO_2_. The cells were transfected with the full-length wild-type PP2Cα and its mutants (D38A and D38K). After 36 hours of transfection and 12 hours of starvation, the HEK293 cells were stimulated with 5 ng/ml EGF for 5 min at 37°C. The levels of ERK1/2 phosphorylation were monitored using a phospho-ERK1/2-pT^202^pY^204^ antibody. Total ERK1/2 was used as a loading control. The western blot shows a representative result from at least three experiments. (b) Statistical analysis of Fig.2a. *: P < 0.05, cells transfected with PP2Cα-WT compared with control cells. #: P < 0.05, cells transfected with D38A or D38K compared with cells transfected with PP2Cα-WT. The data showed are the average of at least three independent experiments. (c) The cellular effects of PP2Cα-WT, D38A and D38K on sorbitol-induced JNK phosphorylation in U251 cells. The U251 cells were cultured in medium similar to the HEK293 cells. 36 hours after transfection, the U251 cells were starved for 12 hours and then stimulated with 0.4 M sorbitol for 30 min at 37°C. The phospho-JNK-pT^221^pY^223^ levels were monitored by western blot. The data shows a representative result from at least three experiments. (d) Statistical graph of Fig.2c. *: P < 0.05, cells transfected with PP2Cα-WT compared with control cells. #: P < 0.05, cells transfected with D38A or D38K compared with cells transfected with PP2Cα-WT. The data showed are the average of three independent experiments at least.

Previous studies have demonstrated that PP2Cα is a potent negative regulator of nerve growth factor (NGF)- or epidermal growth factor (EGF)-induced extracellular regulated protein kinases (ERK) phosphorylation^19^,^22^,^23^ and osmotic stress-induced c-Jun N-terminal kinases (JNK) phosphorylation^24^. ERK and JNK are members of mitogen-activated protein kinases, which are key regulators of multiple signaling pathways. Similar to previous studies, the overexpression of the PP2Cα wild type (PP2Cα-WT) almost abolished EGF-induced ERK1/2 phosphorylation in HEK293 cells (Fig. 2a and 2b). However, the overexpression of an equal amount of PP2Cα-D38A mutant as the wild type only slightly decreased the EGF-induced ERK phosphorylation, and the overexpression of the PP2Cα-D38K mutant has little effect on the ERK phosphorylation increase (Fig. 2a and 2b). Accordingly, the overexpression of the PP2Cα-WT, but not the D38A or D38K mutants, significantly blocked sorbitol-induced JNK phosphorylation in U251 cells (Fig. 2c and 2d). Taken together, these results demonstrated that the D38 mutants impaired PP2Cα-regulated signaling pathways in cells.

### The D38 mutants of PP2Cα decreased the enzyme activity for pNPP, phospho-peptide and phospho-protein substrates

To investigate whether D38 mutants impaired the cellular functions of PP2Cα by affecting its phosphatase activities, we measured the enzyme kinetics of the PP2Cα-WT, D38A and D38K mutants towards the small artificial substrate pNPP, the phospho-peptides derived from pp-ERK (TGFLpT^202^EpY^204^VATR), and pp-P38 (TDDEMpT^180^GpY^182^VAT) (Table 1). For the small artificial substrate pNPP, the *k*_cat_ of D38A and D38K decreased by 2 ~ 4-fold, and the *k*_cat_/K_m_ decreased by approximately 5-fold in comparison with the PP2Cα-WT (Table 1). For the phospho-peptide, the *k*_cat _of D38A and D38K decreased by 3 ~ 45-fold, and the *k*_cat_/K_m_ decreased by 8 ~ 300-fold (Table 1). We further monitored the phosphatase activity of these mutants towards recombinant phospho-ERK protein in the test tube. As shown in Fig.S2, D38 mutants significantly decreased their activity towards the phospho-ERK protein substrate. Taken together, the D38A and D38K mutants of PP2Cα decreased the phosphatase activity toward a panel of small physiological substrates.

### The crystal structures revealed that D38 mutants impaired metal ion binding at the M2 site

**Figure 3:**
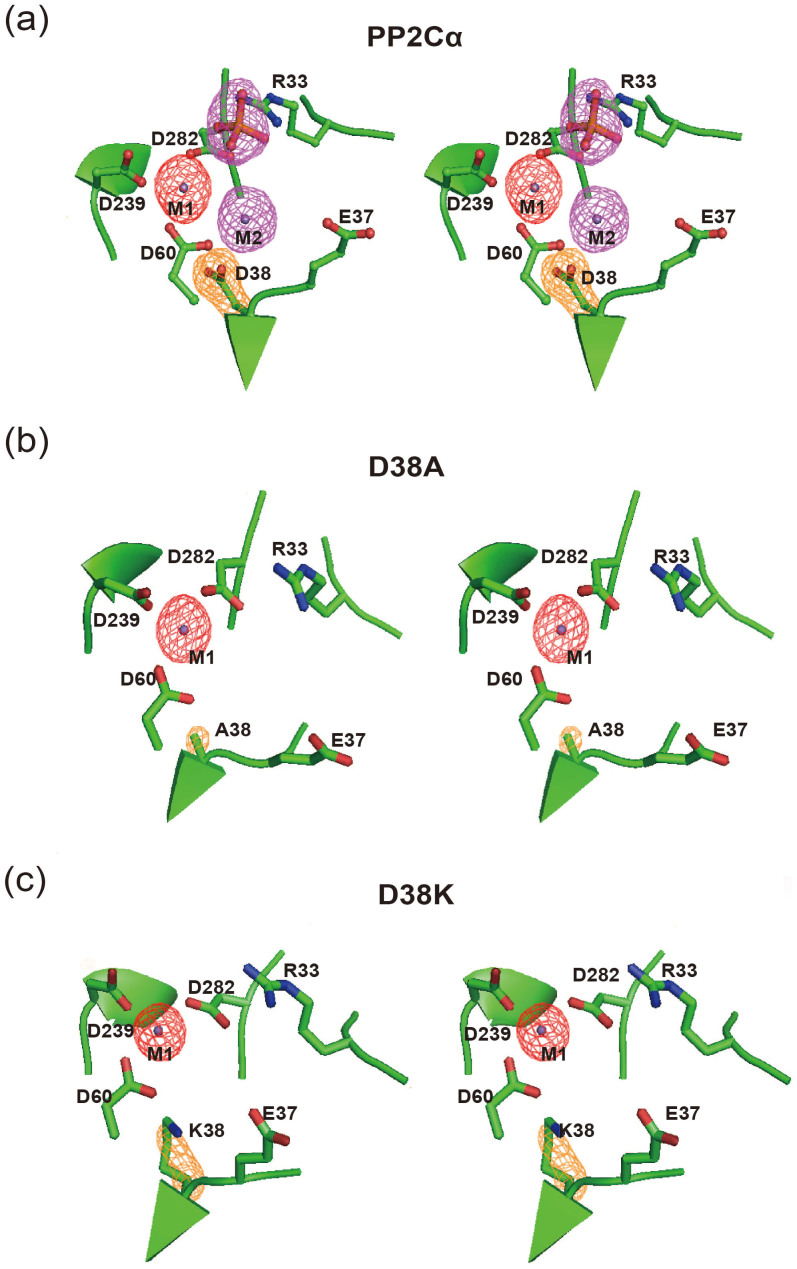
Crystal structures of wild-type PP2Cα and its mutants (D38A and D38K) with Mn^2+^. (a) A stereo view of the unbiased Fo-Fc annealing OMIT map of D38, with two metal ions and phosphate contoured at the 3σ level in the PP2Cα-WT crystal structure. The electron density map of the M1 Mn^2+^, D38, M2 Mn^2+ ^and the phosphate are clearly resolved and are depicted in red, orange and purple, respectively. (b) A stereo view of the unbiased Fo-Fc annealing OMIT map of A38, two metal ion-binding sites and the phosphate binding site contoured at the 3σ level in the PP2Cα-D38A mutants crystal structure. The electron density map of the M1 Mn^2+ ^and A38 are clearly resolved, whereas the electron density for the M2 Mn^2+ ^and phosphate cannot be located. (c) A stereo view of the unbiased Fo-Fc annealing OMIT map of K38, two metal ion-binding sites and the phosphate binding site contoured at the 3σ level in the PP2Cα-D38K mutant crystal structure. The electron density map of the M1 Mn^2+ ^and K38 were mostly resolved, whereas the electron density for the M2 Mn^2+ ^and phosphate were not defined.

To further elucidate the structural basis of the impaired function and catalysis of D38 mutants, we solved the crystal structures of PP2Cα-D38A and D38K with Mn^2+^ at 2.0 Å and 1.85 Å, respectively, and compared these structures with the PP2Cα-WT that was crystallized under similar conditions (1.95 Å) (Supplementary Table S1 and Fig. 3a–c).

In the Fo-Fc-annealing OMIT map, the electron density for the M1 metal ion can be unambiguously assigned in all three PP2C crystal structures. In addition, the electron density for the side chains of D38, A38 and the partial side chain of K38 are also unambiguously assigned in the PP2Cα-WT, PP2Cα-D38A and D38K crystal structures, respectively (Fig. 3a–3c). However, in contrast to the crystal structure of the PP2Cα-WT, the electron density for the M2 metal ion and the phosphate can't be clearly located in the D38A or D38K crystal structures (Fig. 3a–3c).

**Figure 4:**
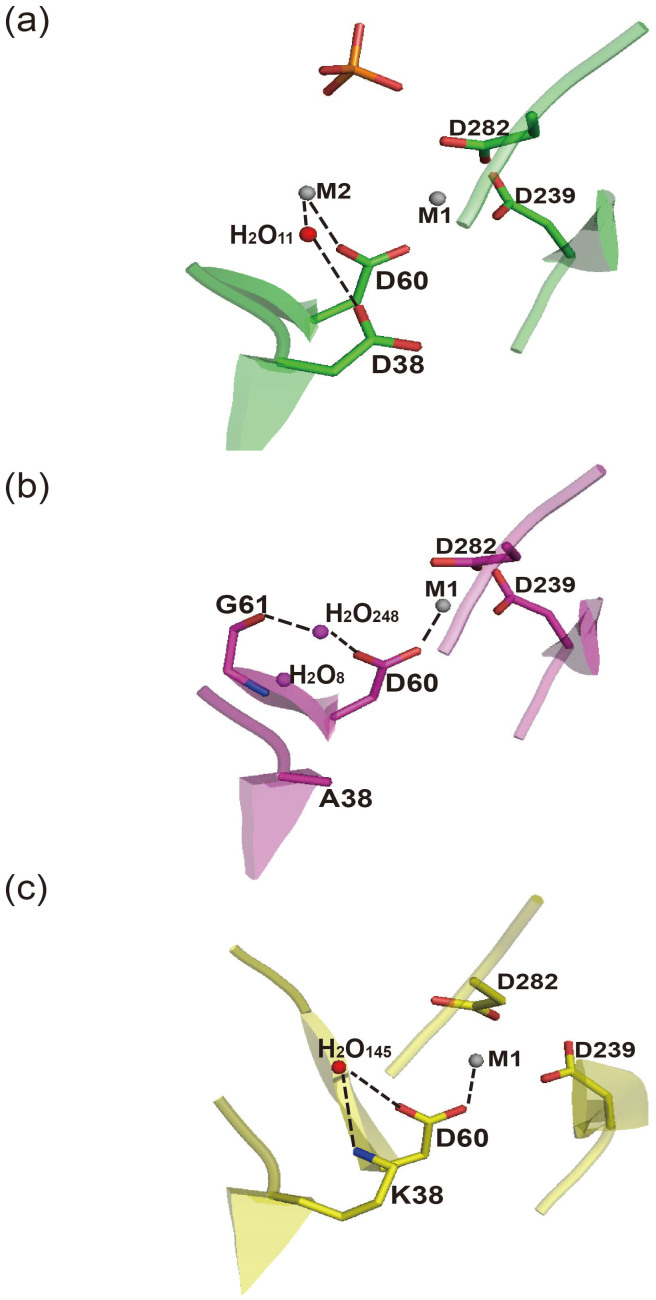
Structure comparisons of the M2 metal ion site of the PP2Cα-WT, PP2Cα D38A and PP2Cα D38K. The hydrogen bond networking and M2 metal ion binding to the M2 metal ion site of the PP2Cα-WT, PP2Cα D38A and PP2Cα D38K are shown.(a) PP2Cα-WT; (b) PP2Cα-D38A; and (c) PP2Cα-D38K.

Specifically, one water (WAT11) between the D38 and D60 in the PP2Cα-WT crystal structure was replaced by two waters (WAT8 and WAT248) in the solvent channel between the A38 and D60 in the D38A crystal structure, which form specific H-bond with the main chain amide of G61 (Fig. 4a–4b and S3a). The positions of these two waters preclude M2 metal ion binding to the D38A mutant protein. In the crystal structure of the D38K mutant, the bridging water between the M2 metal ion and the D38 in the PP2Cα-WT was pushed outward by 0.7 Å because of the longer K38 side chain (Fig. 4a, 4c and S3b). The relocated water (WAT145) forms a hydrogen bond with the side chain of K38, which disabled M2 metal ion binding. Therefore, the crystal structures of PP2Cα-D38 mutants revealed that the D38A or D38K mutants of PP2Cα abolished the M2 metal ion binding by altering the H-bond network of the PP2Cα active site.

### The loss of metal ion binding at the M2 site decreased dianion binding

In the PP2Cα-WT crystal structure, the M2 metal ion forms two water-mediated hydrogen bonds with the oxygen in the phosphate and contributes to substrate dianion binding (Fig. 1a). The loss of the M2 metal ion in the PP2Cα-D38A and D38K mutants may have disturbed the basic nature of the active site and disrupted the water-mediated H-bonds, thus decreasing dianion phospho-substrate binding during intermediate enzyme-substrate formation. Therefore, we measured the inhibition constants of dianion sulfate toward PP2Cα-WT and D38 mutants. Accordingly, the D38A decreased sulfate binding by 4-fold, and D38K decreased the sulfate binding by 10-fold (Table 2). The decreased sulfate binding in D38A and D38K mutants are in agreement with the observation that there are no obvious electron density for phosphate in the crystal structures of D38A and D38K (Fig. 3a–c). Taken together, these results demonstrated that the M2 site metal ion contributes to phospho-substrate binding, which is the initial step of enzyme-substrate complex formation.

### The pH dependence of catalysis revealed that the D38A and D38K mutants affected the rate-limiting chemical step

**Figure 5:**
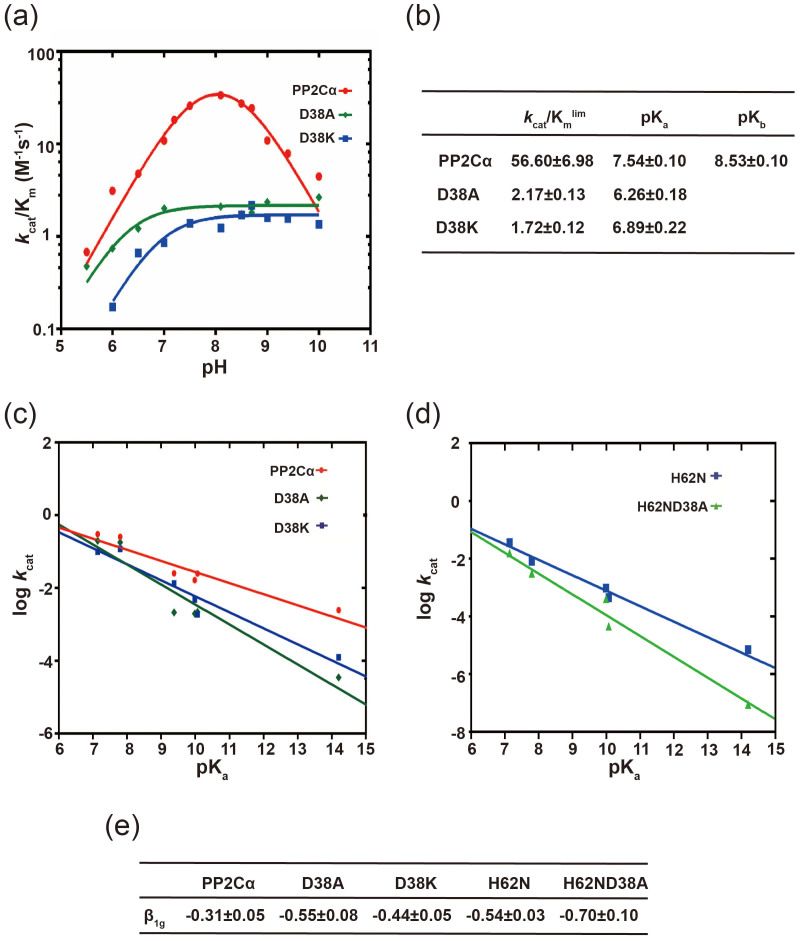
The effects of the pH and the leaving group pK_a_ of substrates on the *k*_cat_/K_m _of wild-type PP2Cα and its mutants. (a) The *k*_cat_/K_m_ values for PP2Cα-WT (red circle), D38A (green diamond), and D38K (blue square) were determined by varying the pNPP concentration at 40 mM Mg^2+^ and different pH values. The assays were performed in Tris/bis-Tris/acetate buffer with different pH values from 5.5 to 10 at 25°C. (b) The pK_a_, pK_b_ and *k*_cat_/K_m_^lim^ values generated from figure 5a. (c) The Brønsted plots for a PP2Cα-catalyzed hydrolysis of aryl phosphates; PP2Cα-WT (red circle), D38A (green diamond) and D38K (blue square). Substrates pNPP (pK_a_ = 7.1), 4-methylumbelliferyl phosphate (pK_a_ = 7.8), β-naphthyl phosphate (pK_a_ = 9.38), phenyl phosphate (pK_a_ = 9.99), O-phospho-L-tyrosine (pK_a_ = 10.07) and phosphoserine (pK_a_ = 14.2) were used. The assays were performed under the same conditions as the pH dependence experiments (Fig. 5a). (d) Brønsted plots for the H62N (blue square) and D38A/H62N double mutations (green triangle). (e) β_1g_ values were obtained by fitting Brönsted plots from figures 5c and 5d.

A bell-shaped curve was observed for the pH dependence of *k*_cat_/K_m_ toward pNPP as catalyzed by PP2Cα-WT in the presence of Mg^2+^ as the activating metal ion, suggesting the presence of the general acid-base catalytic mechanism (Fig. 5a). In consistence with previous studies, the pH dependence of *k*_cat_/K_m_ of PP2Cα-WT toward pNPP indicated that one group must be unprotonated (pK_a_ ~ 7.54) and one group must be protonated (pK_a_ ~ 8.53) (Fig. 5b)^18^,^20^,^21^. Similar to PP2Cα-WT, the *k*_cat_/K_m_ of the D38A and D38K mutants increase from pH 5 to pH 7, indicating the presence of a general base during the catalysis. However, the basic limbs of the D38A and D38K catalyzed pNPP hydrolysis were flat, suggesting an inefficient general acid catalysis for these two mutations.

### The leaving group dependence of the PP2Cα-WT and the D38A and D38K mutants

We therefore further evaluated the effects of the D38 mutants on the general acid catalysis by determining the dependence of the reaction on the nature of the leaving group with a Brønsted plot analysis (Fig. 5c). The β_lg_ values were derived from a linear least-squares fitting of the appropriate Brønsted plots. When the β_lg_ value of the *k*_cat_ is approximately −1, then there is no general acid to stabilize the leaving group of the phenolic oxygen from the substrate. By contrast, when the β_lg_ value of *k*_cat_ is approximately 0, then an efficient general acid is present^25^,^26^,^27^,^28^. As a result, the β_lg_ value of *k*_cat_ decreased from −0.31 for PP2Cα-WT to −0.55 for D38A, and −0.44 for D38K (Fig. 5c and 5e). These results indicate that the D38 mutants developed greater negative charges on the phenolic oxygen of the leaving group in comparison with the PP2Cα-WT.

Previous studies showed that the H62 of PP2Cα might act as a general acid during the cleavage of the P-O bond^21^. In agreement with their findings, the β_lg_ value of *k*_cat_ for H62N was −0.54, suggesting an impaired general acid catalysis. We next measured the β_lg_ value for the *k*_cat_ of the H62N/D38A double mutant to see whether the D38A mutant impaired the general acid catalysis by disturbing the H62 conformation. As a result, the β_lg_ value for the *k*_cat_ of H62N/D38A is −0.7, which is significantly lower than that of both H62N and D38A (Fig. 5d and 5e). Therefore, H62 and D38 independently participate in general acid catalysis, in which they help to provide a potentially positive charged proton to stabilize the negative charge of the oxygen anion after phospho-oxygen bond cleavage. In conclusion, the M2 metal ion plays an important role in stabilizing the negative charge developed by the leaving group of the PP2C substrates.

## Discussion

The catalytic role of the M2 metal ion in PP2C family phosphatases is not defined, partially because of the lack of specific tools for disrupting M2 metal ion binding in the PP2C active site. Both the D38 and E37 of the PP2Cα are conserved among PP2C family members and specifically interact with the M2 metal ion through water-mediated hydrogen bonds (Fig. 1a and 1b). Unlike E37, which is exposed to the solvent, D38 is buried inside the PP2Cα structure and forms an important hydrogen bond with a structural water inside of the protein to stabilize the M2 metal ion binding (Fig. 3a). Our crystal structures revealed that the D38A and D38K mutations altered the water-mediated hydrogen bond network and disrupted M2 metal ion binding with the maintenance of the protein folding and other active site conformations, which provide specific tools to investigate the functional role of the M2 metal ion among PP2C phosphatases (Fig. 3b–c and Supplementary Table S2). Moreover, because the D38 is not surface-exposed, it does not directly participate in substrate hydrolysis.

The binuclear metal center is required for the PP2C family of phosphatases, and the binding of the active site with different metal ions either activates or inhibits the activities of PP2C family phosphatases. However, the catalytic roles for each metal ion remain elusive. In our previous study, we confirmed that cadmium specifically blocked PP2C phosphatase activity by binding to the M1 metal ion site, and the chemical property of the coordinated residue of the M1 metal ion determines the inhibitory specificity of the metal ions^19^. This finding was confirmed by scanning the activating or inhibitory profile of a panel of metal ions towards PP2Cα and PP2Cγ, together with an analysis of a series of PP2C mutants. These results suggested that the M1 metal ion is one determinant for catalysis. Using specific M2 metal ion-coordinated residue D38A and D38K mutations, we discovered that the M2 metal ion participates in the initial step of dianion phospho-substrate binding, affects the rate-limiting chemical step and is involved in the neutralization of the negative charge in the leaving group according to a combined enzymology analysis. Interestingly, the D38A and the previously proposed general acid known as H62 work independently to stabilize the leaving group as revealed by Brönsted plot analyses. It is likely that both H62 and the M2 metal ion-coordinated water can function as general acids during catalysis.

Besides the binuclear metal center, recent studies demonstrated that a third metal ion is required for the catalysis of PP2Cα and PP2Cδ toward phospho-peptide substrates^29^. Biochemical studies and mutating effects suggest that the third metal ion M3 interact with residues of D146, D243 and D239 of PP2Cα. However, the exact mechanism of M3 metal ion participating in PP2Cα catalysis is still lacking and the third metal ion is missing in the crystal structures of PP2Cα wild type or mutants due to the low pH of crystallization condition. Further structural and enzymologic studies are required to fully understand the catalytic roles of metal ions in the PP2C family phosphatases.

Taken together, we have identified the specific catalytic roles of the M2 metal ion of PP2C family phosphatases; these roles include dianion binding during enzyme-substrate complex formation and the stabilization of the leaving group after the cleavage of the phospho-aryl oxygen bond. In addition to the newly characterized roles of the M2 metal ion during catalysis, there could be a general mechanism that is used by other metalloenzymes that provides clues for the design of new PP2C inhibitors and de-novo enzymes.

## Methods

### Materials

Para-nitrophenyl phosphate (pNPP), β-naphthyl phosphate, phenyl phosphate, 4-methylumbelliferyl phosphate and polyethylene glycol (PEG mol. wt. 8000) were purchased from Sangon Biotech Co., Ltd. (Shanghai, China). O-phospho-L-tyrosine was purchased from Sigma. Peptides including ppERK2 (TGFLpT^202^EpY^204^VATR) and pp-P38 (TDDEMpT180GpY182VAT) were obtained from China Peptides Co. (China). The BIOMOL green TM reagent for phosphate detection (BML-AK111) was purchased from Enzo Life Sciences. The Flag tag (DYKDDDDK) antibody, GADPH antibody, pp-P38 MAPK (pT^180^/pY^182^) antibody and pp-P44/42 MAPK (ERKpT^202^/pY^204^) antibody were purchased from Cell Signaling Technology. Ni-NTA agrose was purchased from Roche. All other materials are from Sigma company. All other materials are from Sigma company.

### Constructs and site-directed mutagenesis

The full-length human PP2Cα cDNA was a gift from Professor Patricia T. W. Cohen (MRC Protein Phosphorylation Unit). The Flag-tagged PP2Cα construct used for the cellular assays was described previously. The PP2Cα mutants (D38A, D38K, H62N and H62N/D38A) were generated with a QuickChange mutagenesis kit. PAGE-purified oligonucleotide primers were purchased from the Beijing Genomics Institute (China) and all mutations were verified by DNA sequencing.

### Protein purification

The expression and purification of PP2Cα with an N-terminal His tag in addition to its mutants have been described previously^19^,^22^. In brief, BL21 *E. coli* cells were transformed with PP2Cα plasmids and cultured at 37°C. The cultures were induced with 0.5 mM IPTG overnight at 25°C and then pelleted by centrifugation at 3,200 g. The cell pellets were re-suspended in 60 ml of ice-cold “His buffer” (20 mM Tris, pH 8.0, and 300 mM NaCl) and crushed three times with a high-pressure cell cracker. The lysates were then centrifuged at 13,000 g for 40 min at 4°C. The supernatant was incubated with 1 ml of Ni beads (Ni-NTA agrose) for 1.5 h. The Ni beads were then washed with 100 ml of ice-cold “His buffer” at 4°C. The bound His-PP2Cα protein was finally eluted with a buffer containing 20 mM Tris pH 8.0, 300 mM NaCl and 100 mM imidazole. Before being frozen at −80°C, the proteins were exchanged for a buffer containing 0.05 M Tris, 0.05 M bis-Tris, 0.1 M acetate, pH 8.0, 1 mM DTT and 1 mM Mn^2+^. The purity of the protein was examined by Coomassie brilliant blue staining after SDS-PAGE. The protein concentration was measured at OD_260_/OD_280_ in a spectrophotometer^30^. The molar absorption coefficient of PP2Cα at 280 nm (ε_280_) is 36035 M^−1^cm^−1^ according to the following equation 1^30^.
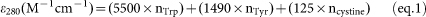
n_Trp_: number of Trp residues, n_Tyr_: number of Tyr residues, n_cystine_: number of disulfide bonds in the protein.

For purification of PP2Cα without an N-terminal His tag, the BL21 E.coli was harvested by centrifugation at 3,200 g for 20 min, and then re-suspended in 20 ml of buffer A (50 mM Tris-HCl pH 7.5, 1 mM DTT, 2 mM MnCl_2_, 1 mM EDTA, 0.1 mM EGTA, 100 mM NaCl, 0.1 mM phenylmethylsulfonyl fluoride, 0.03% (v/v) Brij-35). The bacteria solution was crushed by high pressure cell cracker three times, and then the lysate was centrifuged at 17,000 g for 20 min at 4°C. The supernatant was precipitated with 33% and 50% (NH_4_)_2_SO_4_ (w/v) twice. After centrifugation at 17,000 g for 40 min, the pallet was re-suspended by buffer B (25 mM Na_2_HPO_4_ pH7.0, 1 M (NH_4_)_2_SO_4_, 1 mM DTT, 2 mM EDTA). Then the supernatant was running through a hydrophobic interaction chromatography and a Q-Sepharose. The peaks with phosphatase activity were pooled and concentrated, then was further purified by sieve chromatography after dialyzing against 1 L of sieve buffer (50 mM Tris-HCl pH7.0, 150 mM NaCl, 1 mM DTT) for 2 h at 4°C. At last, the protein was collected and concentrated to 15 mg/ml stored at −80°C.

### Crystallization

Crystallizations of PP2Cα protein were performed as previously described^18^. Briefly, PP2Cα protein without N-terminal His tags (wild type, D38A and D38K) were concentrated to 15 mg/ml before crystallization. Crystallizations were performed by vapor diffusion at 4°C, and the ratio of protein to precipitating solution (50 mM potassium phosphate, pH 5.5, 10% (w/v) PEG8000, 15% (v/v) glycerol and 2 mM DTT) was 1:2. Seeding was occasionally performed to achieve large crystals that were suitable for diffraction.

### Data collection and structure determination

Diffraction data were collected in Shanghai Synchrotron Radiation Facility (BL17U). Data for PP2Cα (wild type, D38A and D38K) were processed using the HKL2000 program and their structures were determined by molecular replacement with Phaser^31^ in the CCP4 software package. The crystals all belong to the P3_1_2_1_ space group. A single chain of the PP2Cα (PDB code 1A6Q, water deleted) was used as the initial search model^32^,^33^. Further refinements of the crystals were processed by the PHENIX program with iterative manual building in COOT^33^. To reduce the phase biases during further refinement, composite omitted maps were built and density modification was executed. Ramachandran plots were calculated with PROCHECK and COOT. The statistics for the final solved structures are shown in Table S1.

### Cell culture, transfection, and western blotting

Human kidney 293 cells and U251 cells were cultured in DMEM including 1% penicillin/streptomycin, 10% fetal bovine serum and 25 mM glucose under a humidified atmosphere containing 5% CO_2_ at 37°C. HEK293 and U251 cells were transfected with the full-length wild-type PP2Cα and its mutants (D38A and D38K) with an N-terminal Flag tag with PEI (Polysciences). Thirty-six h after the transfection, the cells were starved for 12 h and then stimulated with 5 ng/ml EGF for 5 min (HEK293 cells) and 0.4 M sorbitol for 30 min (U251 cells) at 37°C. The cells were subsequently washed twice with cold phosphate-buffered saline (PBS) and then harvested with lysis buffer (50 mM Tris-HCl, pH 7.5, 150 mM NaCl, 0.25% (m/v) sodium deoxycholate, 1% NP-40, 1 mM EDTA, 50 mM NaF, 10% (v/v) glycerol, 1 mM Na_3_VO_4_, 5 mM iodoacetamide (IAA) and protease inhibitor cocktail). As an irreversible peptidases inhibitor, IAA can react with sulfhydryl of cysteine residues and prevent the formation of disulfide bonds. The cells were lysed on ice for 30 min and then centrifuged at 13,000 g for 30 min at 4°C to collect the supernatant. The protein sample concentrations were measured with a BCA Protein Quantitation Kit. The same amount of 2 × SDS loading buffer was added to lysate proteins and then boiled for 10 min. The protein samples were then subjected to western blotting. Proteins were transferred to nitrocellulose membranes after running SDS-PAGE and incubated in appropriate primary antibodies at 4°C overnight, and then incubated in HRP-conjugated secondary antibody. Reactive bands were detected with Western Chemiluminescent HRP Substrate (Millipore). The ImageJ software was used to quantify the signals from western blots and a standard curve of Western blott with linear increase of the target protein amount was used to correct the potential deviations.

### Enzyme Kinetics

To determine the kinetic parameters of *k*_cat_ and *k*_cat_/K_m_, the reactions were performed in a reaction buffer (0.05 M Tris, 0.05 M bis-Tris, and 0.1 M acetate pH 7.0) containing suitable divalent Mn^2+^ at 25°C. The initial velocities were measured at appropriate pNPP concentrations and saturating levels of Mn^2+^. The reactions were terminated by 500 mM EDTA (pH 10.0) and assessed by measuring the absorbance of pNP at 405 nm. For enzyme assays using phospho-peptides as substrates, an inorganic phosphate assay with an end-point reading at 620 nm was determined for phosphate release. The K_m_, *k*_cat_ and *k*_cat_/K_m_ values were obtained by fitting the data to equation 2.
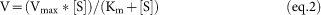
To measure the pH dependence of phosphatase catalysis, the *k*_cat_ and *k*_cat_/K_pNPP_ of PP2Cα (wild-type, D38A and D38K) were obtained at various pH values (Bis-Tris/Tris-acetate buffer) with pNPP as the substrate at a saturating Mg^2+ ^level (40 mM for PP2Cα). The pH data were fitted to Equation 3 and Equation 4. ((*k*_cat_/K_m_)^lim^ means the pH-independent value of *k*_cat_/K_m_, K_a_ and K_b_ are the apparent ionization constants of the enzyme-substrate complex in the rate-limiting step, and [H] is the concentration of H^+^ ions.)
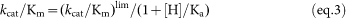

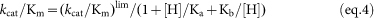


To measure the leaving group dependence for the PP2C phosphatase-catalyzed reaction, the dephosphorylation rates were analyzed for small artificial substrates with different pK_a_ values as previously described^20^. The *k*_cat_ towards substrates (pNPP (7.1), 4-methylumbelliferyl phosphate (7.8), β-naphthyl phosphate (9.38), phenyl phosphate (9.99), o-phospho-L-tyrosine (10.07) and phosphoserine (14.1)) were analyzed using the inorganic phosphate detection assay at pH 7.0. The log(*k*_cat_) values were plotted against the pK_a_ of leaving-group of the substrates to acquire the Brönsted plots. The β_lg_ values for *k*_cat_ were obtained from fitting the data to a linear least-square regression equation.

### The in vitro dephosphorylation of phospho-ERK2 protein

The phosphorylation of His-tagged ERK2 protein *in vitro* was performed as previously described^34^,^35^. 1 mg/ml purified His-tagged ERK2 protein and 0.1 mg/ml purified His-tagged MEK1/G7B protein were added into a 500 μl reaction system in a reaction buffer (10 mM Hepes pH 7.4, 20 mM Mg(COOH)_2_, 100 mM NaCl, 0.5 mM ATP and 2 mM DTT) for in vitro phosphorylation. After incubated at 30°C for 90 min, the phospho-ERK2 protein was separated from free ATP through a Superdex-200 column with FPLC. Then the phosphorylation was confirmed by mass spectrometry and the protein was concentrated and stored at −80°C.

The dephosphorylation of phospho-ERK protein by PP2Cα-WT and its mutants (D38A, D38K) was performed in a reaction buffer containing 0.05 M Tris, 0.05 M bis-Tris, 0.1 M acetate and saturated Mn^2+^ at 37°C, pH 7.0 as previously described. The reactions were terminated by adding 10 mM EDTA and loading buffer, followed by boiling in SDS buffer at 100°C for 10 min. ERK dephosphorylation was analyzed by western blotting with a specific anti-ppERK-pT^202^pY^204^ antibody. The gel bands were quantified by Image J software.

### SO_4_^2-^ inhibition assay

The K_i_ values for the inhibition of PP2Cα-WT and its mutants by SO_4_^2−^ were performed using pNPP as the substrate in the buffer (0.05 M Tris, 0.05 M bis-Tris and 0.1 M acetate at 25°C, pH 7.0, and 10 mM Mn^2+^) with varying concentrations of pNPP and Na_2_SO_4_. The data were fitted to equation 5.
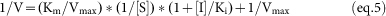


### Data analysis

All data were presented as the means ± SEM. Statistical comparisons were made with one- or two-way ANOVA tests in GraphPad Prism5. A multiple sequence alignment was performed in T-coffee. The western blots were scanned, and the gel band intensity was quantified with Image J software (National Institutes of Health, Bethesda MD). All protein-structure descriptions were generated in PyMOL (http://www.pymol.org).

## Supplementary Material

Supplementary Information Supplementary Material for: The catalytic role of the M2 metal ion in PP2Cα

## Figures and Tables

**Table 1:** Kinetic constants of the activity of PP2Cα and its mutants at D38 position (D38A and D38K) toward pNPP and phospho-peptides

	pNPP			
	*k*_cat_ (s^−1^)	K_m_ (mM)	*k*_cat_/K_m_ (M^−1^s^−1^)	Ratio of *k*_cat_/K_m_ (wild-type/mutant)
**PP2Cα**	2.52 ± 0.06	4.05 ± 0.25	622 ± 23	1
**D38A**	1.46 ± 0.05	12.2 ± 1.08	120.3 ± 8.7	5.17
**D38K**	0.68 ± 0.03	4.88 ± 0.68	139 ± 15	4.49

	ERK-pT202pY204			
	[ phospho-peptides derived from pp-ERK (TGFLpT^202^EpY^204^VATR) ]			
	*k*_cat_ (s^−1^)	K_m_ (μM)	*k*_cat_/K_m_ (10^4^ M^−1^s^−1^)	Ratio of *k*_cat_/K_m_ (wild-type/mutant)
**PP2Cα**	0.36 ± 0.03	5.5 ± 1.1	6.55 ± 1.46	1
**D38A**	0.12 ± 0.01	15.8 ± 2.0	0.76 ± 0.29	8.62
**D38K**	0.008 ± 0.0002	37.5 ± 6.9	0.02 ± 0.002	327.5

	P38-pT180pY182			
	[ phospho-peptides derived from pp-P38 (TDDEMpT^180^GpY^182^VAT) ]			
	*k*_cat_ (s^−1^)	K_m_ (μM)	*k*_cat_/K_m_ (10^4^ M^−1^s^−1^)	Ratio of *k*_cat_/K_m_ (wild-type/mutant)
**PP2Cα**	0.26 ± 0.03	23.9 ± 4.1	1.09 ± 0.20	1
**D38A**	0.08 ± 0.01	31.4 ± 6.0	0.25 ± 0.07	4.36
**D38K**	0.002 ± 0.0004	17.7 ± 2.6	0.01 ± 0.0024	109

The assays were performed in the Bis-Tris/Tris-acetate buffer (0.05 M Tris, 0.05 M Bis-Tris, 0.1 M acetate) pH 7.0 containing 10 mM Mn^2+^ and 1 mM DTT at 25°C. The *k*_cat_, K_m_ and *k*_cat_/K_m _values were fitted to the equation 2 with GraphPadPrism5 software. The data were repeated thrice at least.

The assays were performed in the Bis-Tris/Tris-acetate buffer (0.05 M Tris, 0.05 M Bis-Tris, 0.1 M acetate) pH 7.0 containing 10 mM Mn^2+^ and 1 mM DTT at 25°C. The *k*_cat_, K_m_ and *k*_cat_/K_m _values were fitted to the equation 2 with GraphPadPrism5 software. The data were repeated thrice at least.

The assays were performed in the Bis-Tris/Tris-acetate buffer (0.05 M Tris, 0.05 M Bis-Tris, 0.1 M acetate) pH 7.0 containing 10 mM Mn^2+^ and 1 mM DTT at 25°C. The *k*_cat_, K_m_ and *k*_cat_/K_m _values were fitted to the equation 2 with GraphPadPrism5 software. The data were repeated thrice at least.

**Table 2:** Inhibition constant of SO_4_^2−^ toward PP2Cα, D38A and D38K

	PP2Cα	D38A	D38K
**SO_4_**^2^^−^ **K_i_ (mM)**	15.4 ± 1.3	61.8 ± 5.1	213.2 ± 56.8

The assays were performed in the buffer containing 0.05 M Tris, 0.05 M Bis-Tris, 0.1 M acetate and 10 mM Mn^2+^ at 25°C and pH 7.0 with varying concentrations of pNPP and Na_2_SO_4_. The data were fitted to the equation 5 and were analyzed by software GraphPadPrism5. The data showed were repeated three times at least.
