# Supplementary material for: The catalytic role of the M2 metal ion in PP2Cα
Source: Sci Rep. 2015 Feb 24;5:8560. doi: 10.1038/srep08560 (PMC5390078; doi:10.1038/srep08560)
Supplement: Supplementary Information — Supplementary Material for: The catalytic role of the M2 metal ion in PP2Cα [file srep08560-s1.doc]

Supplementary Material for:

**The catalytic role of the M2 metal ion in PP2Cα**

Chang Pan^1,2#^, Jun-yi Tang^3,4#^, Yun-fei Xu^1,2^, Peng Xiao^1,3^, Hong-da-Liu^1,3^, Hao-an Wang^5^, Wen-bo Wang^1,3^, Fan-guo Meng^6^, Xiao Yu^3,4*^, Jin-peng Sun^1,3,7*^

1.Key Laboratory Experimental Teratology of the Ministry of Education and Department of Biochemistry and Molecular Biology, Shandong University, School of Medicine, Jinan, Shandong, China

2. Qilu Hospital of Shandong University, Jinan, China

3. Shandong Provincial School Key laboratory for Protein Science of Chronic Degenerative Diseases, Jinan, Shandong, China

4. Department of Physiology, Shandong University, School of Medicine, Jinan, Shandong, China

5. Department of Human Biology, University of Toronto, Toronto, Ontario, Canada

6. Yangtze Delta Region Institute of Tsinghua University, Zhejiang, China

7. Provincial Hospital affiliated to Shandong University, Jinan, Shandong, China

* Corresponding author: Xiao Yu (corresponding author)

Email: [yuxiao@sdu.edu.cn](mailto:yuxiao@sdu.edu.cn)

Jin-Peng Sun (corresponding author)

Email: [sunjinpeng@sdu.edu.cn](mailto:sunjinpeng@sdu.edu.cn)

*Web:* *http://www.yuxiao-sunjinpeng-lab.org/Published_Papers/*

^#^ Authors contributed equally to this work.

**This file includes:**

**SUPPLEMENTARY TABLES**

**Supplementary Table S1:** Data collection and refinement statistics.

**Supplementary Table S2:** Translocation distances of several amino acids in crystal structures of D38A and D38K compared with crystal structure of PP2Cα-WT (unit: Å).

**SUPPLEMENTARY FIGURES**

**Supplementary Fig. S1:** Multiple sequence alignment of human PP2Cα with the human PP2Cβ, *Saccharomyces cerevisiae* PTC2, *Arabidopsis thaliana* PP2CA, *Arabidopsis thaliana* ABI1, *Arabidopsis thaliana* HAB1, *Saccharomyces cerevisiae* adenyl cyclase and *Bacillus subtilis* SpollE.

**Supplementary Fig. S2:** The dephosphorylation of phospho-ERK by PP2Cα-WT, D38A and D38K mutants.

**Supplementary Fig. S3:** Overlays of the crystal structures of the wild-type PP2Cα with the PP2Cα D38 mutants.

**CONTENTS：**

**Supplementary Table S1. Data collection and refinement statistics.**

|  | **PP2Cα-WT** | **D38A** | **D38K** |
| --- | --- | --- | --- |
| Data Collection |  |  |  |
| Space group | p3_1_2_1_ | p3_1_2_1_ | p3_1_2_1_ |
| Cell Dimensions |  |  |  |
| a (Å) | 90.71 | 91.26 | 90.82 |
| b (Å) | 90.71 | 91.26 | 90.82 |
| c (Å) | 105.44 | 105.767 | 105.41 |
| α (deg) | 90 | 90 | 90 |
| β (deg) | 90 | 90 | 90 |
| γ (deg) | 120 | 120 | 120 |
| Resolution (Å) | 80 – 1.95(2.02 – 1.95) ^a^ | 80 – 2.0(2.1-2.0) ^a^ | 80 – 1.85(1.88-1.85) ^a^ |
| Unique observations | 37500(3688) ^a^ | 34825(3439) ^a^ | 43274(2127) ^a^ |
| Completeness (%) | 99.9(100)^a^ | 99.8(99.8)^a^ | 99.7(99.9)^a^ |
| Redundancy | 5.5(5.6)^a^ | 9.3(9.2)^a^ | 4.4(3.6)^a^ |
| <I>/<σ> | 45.64(6.45)^a^ | 54.05(11.48)^a^ | 80.42(11.43)^a^ |
| R_merge_ | 0.075(0.563)^a^ | 0.081(0.612)^a^ | 0.074(0.663)^a^ |
| Structure Refinement | 80 – 1.95 | 80 – 2.0 | 80 – 1.85 |
| Resolution (Å) | 1.95 | 2.00 | 1.85 |
| Reflections used for R_work_/R_free_ |  |  |  |
| R_work_ /R_free_^b^ (%) | 0.1957/ 0.2235 | 0.1943/0.2223 | 0.1991/ 0.2297 |
| Average B-factor |  |  |  |
| Protein | 32.31 | 25.3 | 37.02 |
| Metal | Mn^2+^ | Mn^2+^ | Mn^2+^ |
| RMSD ideal bonds (Å) | 0.007 | 0.007 | 0.006 |
| RMSD ideal angles (deg) | 1.091 | 1.131 | 1.103 |
| Ramachandran Plot (%) |  |  |  |
| Most favored | 0.9471 | 0.9469 | 0.9581 |
| Allowed | 0.0334 | 0.0335 | 0.0307 |
| Generously allowed | 0.0195 | 0.0196 | 0.0112 |
| Disallowed | 0 | 0 | 0 |

^a^ Each dataset was collected from a single crystal.

The values in parentheses correspond to the highest resolution shell.

**Supplementary Table S2: Translocation distances of several amino acids in crystal structures of D38A and D38K compared with the crystal structure of PP2Cα-WT (unit: Å).**

|  | **D38A** | **D38K** |
| --- | --- | --- |
| **E37** | 0.21 | 0.64 |
| **M36** | 0.36 | 0.34 |
| **H40** | 0.83 | 0.75 |
| **D60** | 0.22 | 0.25 |
| **G61** | 0.26 | 0.22 |
| **H62** | - | 0.18 |
| **D282** | 0.17 | 0.2 |
| **D239** | 0.23 | - |

**Supplementary Fig. S1:** Multiple sequence alignment of human PP2Cα with the human PP2Cβ, *Saccharomyces cerevisiae* PTC2, *Arabidopsis thaliana* PP2CA, *Arabidopsis thaliana* ABI1, *Arabidopsis thaliana* HAB1, *Saccharomyces cerevisiae* adenyl cyclase and *Bacillus subtilis* SpollE.

**
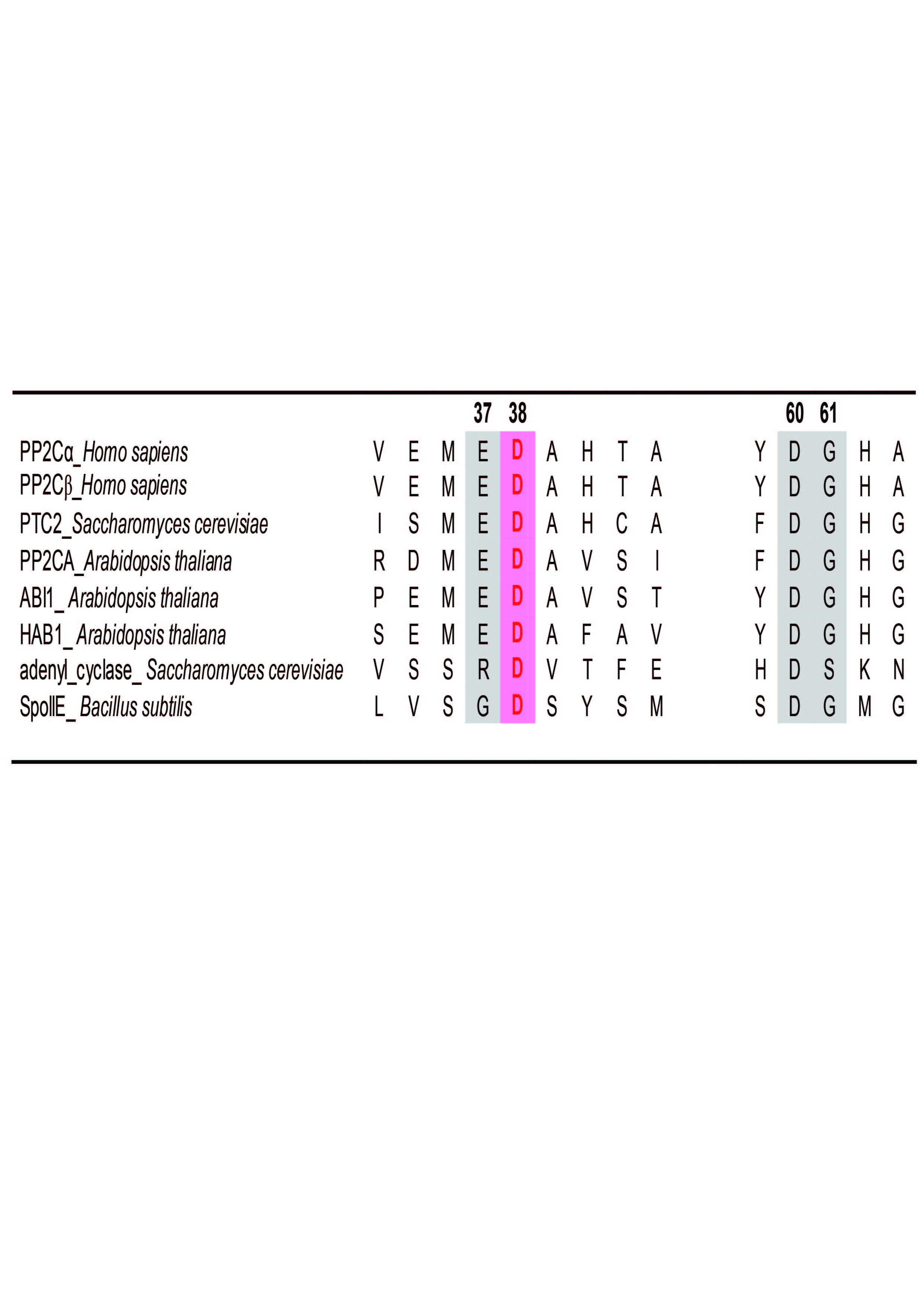
**

The 38^th^ amino acid residue D of PP2Cα and the corresponding residues of other phosphatases are marked red. Residues related to the M2 binding site are shadowed.

**Supplementary Fig. S2** The dephosphorylation of phospho-ERK protein substrate by PP2Cα-WT, D38A and D38K mutants


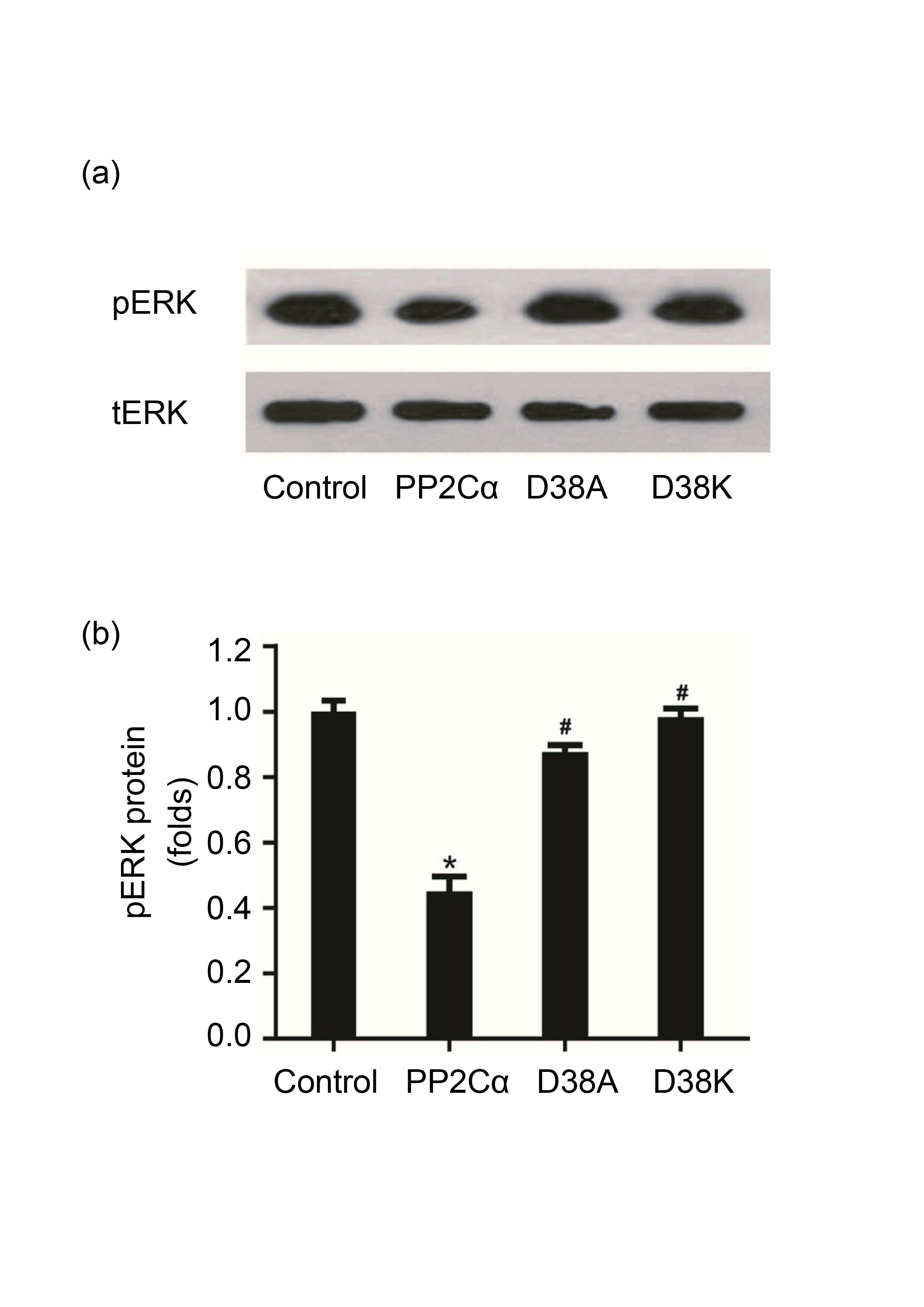


(a) Representative western blot of the activity of PP2Cα-WT, D38A and D38K mutants toward phospho-ERK protein substrate

(b) statistical analysis of the data from (a). The level of ERK phosphorylation was determined with a specific phospho-ERK antibody. *, P<0.05; compared with the control. #: P<0.05; the activity of D38A or D38K mutants were compared with the wild-type PP2Cα. The data displayed are the average of at least three independent tests.

**Supplementary Fig. S3.** Overlays of the crystal structures of the wild-type PP2Cα with the PP2Cα D38 mutants


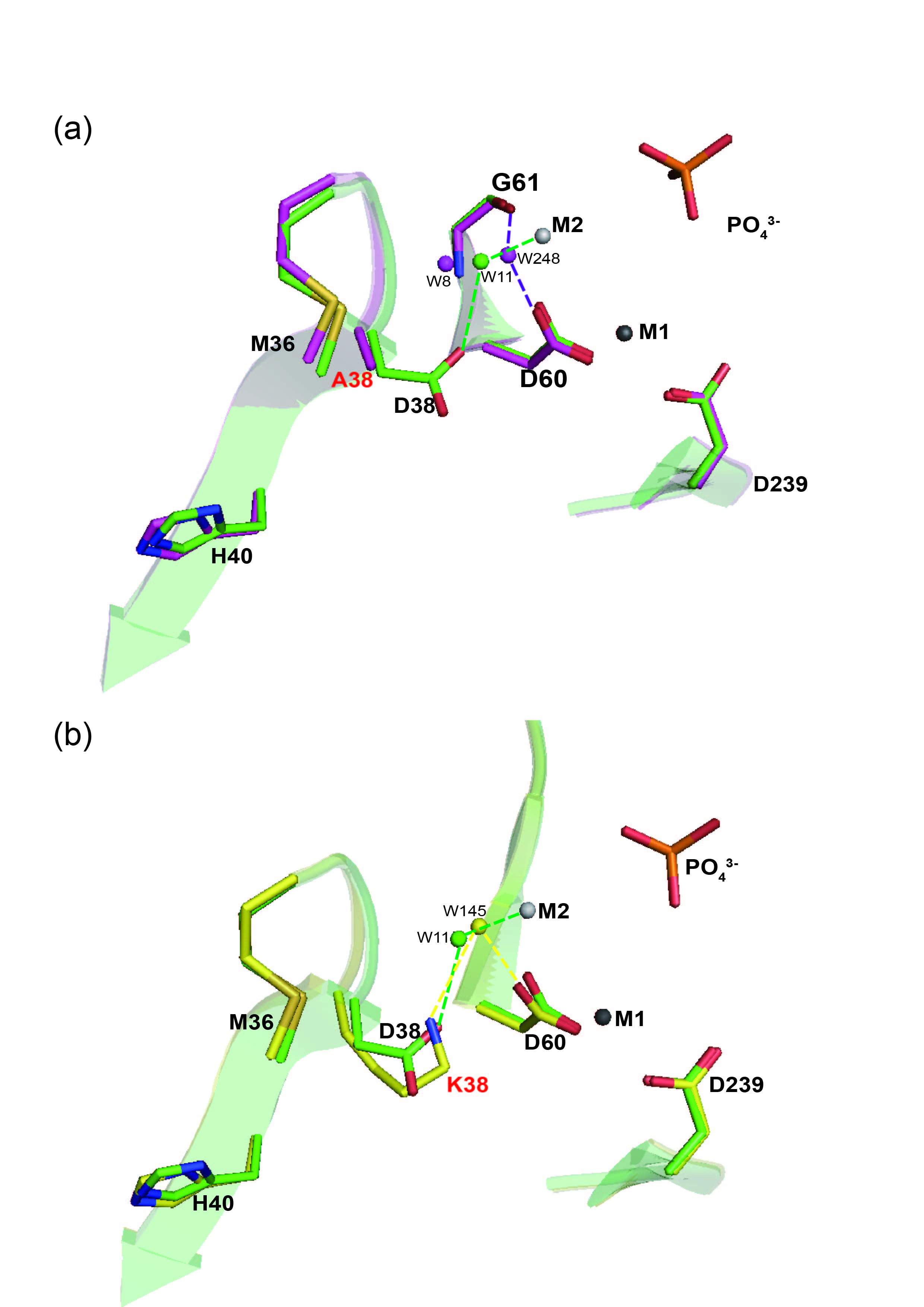


Superposition of the crystal structures of the wild-type PP2Cα with D38A (a) and D38K (b). The specific residues are shown as stick-ball mode. The wild-type PP2Cα, D38A and D38K are shown in green, purple and yellow, respectively. H-bonds are showed as dotted line. H-bonds between water and metal ion or amino acid of wild-type PP2Cα, D38A and D38K are displayed in green, purple and yellow, respectively. M1 metal ion is dark gray and M2 metal ion is light gray.
